# Emotional Analysis of Twitter Posts During the First Phase of the COVID-19 Pandemic in Greece: Infoveillance Study

**DOI:** 10.2196/27741

**Published:** 2021-09-29

**Authors:** Styliani Geronikolou, George Drosatos, George Chrousos

**Affiliations:** 1 Biomedical Research Foundation of the Academy of Athens Athens Greece; 2 University Research Institute of Maternal and Child Health and Precision Medicine Athens Greece; 3 Institute for Language and Speech Processing, Athena Research Center Xanthi Greece

**Keywords:** emotional analysis, COVID-19, Twitter, Greece, infodemics, emotional contagion, epidemiology, pandemic, mental health

## Abstract

**Background:**

The effectiveness of public health measures depends upon a community’s compliance as well as on its positive or negative emotions.

**Objective:**

The purpose of this study was to perform an analysis of the expressed emotions in English tweets by Greek Twitter users during the first phase of the COVID-19 pandemic in Greece.

**Methods:**

The period of this study was from January 25, 2020 to June 30, 2020. Data collection was performed by using appropriate search words with the filter-streaming application programming interface of Twitter. The emotional analysis of the tweets that satisfied the inclusion criteria was achieved using a deep learning approach that performs better by utilizing recurrent neural networks on sequences of characters. Emotional epidemiology tools such as the 6 basic emotions, that is, joy, sadness, disgust, fear, surprise, and anger based on the Paul Ekman classification were adopted.

**Results:**

The most frequent emotion that was detected in the tweets was “surprise” at the emerging contagion, while the imposed isolation resulted mostly in “anger” (odds ratio 2.108, 95% CI 0.986-4.506). Although the Greeks felt rather safe during the first phase of the COVID-19 pandemic, their positive and negative emotions reflected a masked “flight or fight” or “fear versus anger” response to the contagion.

**Conclusions:**

The findings of our study show that emotional analysis emerges as a valid tool for epidemiology evaluations, design, and public health strategy and surveillance.

## Introduction

Emotional involvement in health care and disease has been subjected to medical evaluation since antiquity [1]. *Humorism* (or Humoralism) was a theory implemented by Hippocrates [2] and coined by Galen; this theory classifies the basic emotions as well as their impact on health and disease [1,2]. Further, in the Hippocratic Collection (“*Corpus Hippocraticum”*) [3], communicable diseases were discussed. The historian Thucydides described the Athenian “*plague*”—a contagious pandemic flow of uncertain etiology, perhaps typhoid fever [4,5]—which originated from Ethiopia and was transmitted to the Athenian population during the Peloponnesian War (around 430 BC) [6,7]. Since then, humankind has faced numerous contagious disease epidemics of varying time spans. During all eras, under different societal circumstances, citizens interpreted the shocking reality of epidemics in similar ways: they expressed basic emotions such as stress, fear, and anger [8]. These emotions were intertwined with the epidemic contagion. Thucydides narrated that citizens’ panic made them often indifferent to legal, moral, hygienic, or religious rules—a phenomenon described as “*acedia*” [5,8]. Similarly, in several epidemics of plagues in Central Europe during the Middle Ages, collective emotions included fear, anger, and indifference to public health measures, with negative societal and political consequences.

The “Spanish flu” or influenza of the 1918 pandemic, wherein the fatality rate in Greece was as high as 0.33%, was the last time that the Greeks experienced societal isolation measures [9]. More recent epidemics such as those of SARS-CoV (2003), *West Nile virus* (2010-2011), or HIV (2011) did not really affect Greece, as in the first case, the virus did not prevail in the country; in the second, the incidence was extremely low; and in the third, it was limited to a specific population of drug users [10]. Furthermore, in the published literature, we cannot find Greek-specific reports focusing on the emotional impact of these epidemics. Similarly, the H1N1 epidemic impact on Greek general population was not investigated, and the health care providers’ worries about the safety of their families were not recorded [11,12]. A recent study linked temperament or psychopathology with the effectiveness of public health measures [13], while another study linked morality to public trust and efficacy of public health measures [14]. These recent approaches implicated “thinking” to emotions—a state that is associated with fundamental emotions such as fear, joy, and surprise. Thus, to study basic emotions rather than others that come after or are more complex or include rational processing is a priority.

Analyzing the general emotions of the population during the current pandemic is a *sine qua non* for the effectiveness of public health planning and application of prevention measures. This has been evidenced by experiments [15,16] and real data [17]. The COVID-19 pandemic due to the SARS-CoV-2 virus has occurred at a time when technology offers opportunities to use social media for business, human communication, or pleasure. Associate Professor Heidi Tworek at the University of British Columbia stressed on Twitter that “Communications in a public health crisis are as crucial as medical interventions …. in fact, communication policies are a medical intervention” [18,19]. Using the cascade of information flowing from social media is of retrospective, real-time, and future value for epidemiology analysis. Interdisciplinary work and collaborations are of major value and much needed, and this has been confirmed in the most prominent way during the current sanitary crisis. In a previous work, we suggested that basic emotional reactivity—as expressed in social media—is ethnicity/culture-dependent [17].

As for the COVID-19 pandemic, although sparse surveys targeting the Greek population (general or health care providers) have been published [10,20,21], none of them evaluated social media data. In contrast, during this pandemic, social media messages have been emotionally evaluated in several countries such as Italy, Iraqi Kurdistan, Korea, United States of America, and China [17]. In fact, Twitter-focused emotional analysis studies have been evaluated in more than 170 countries [17,22,23]. As the emotional evaluation of the tweets in Greece does not exist, this work attempted to fill this knowledge gap by studying the tweets posted during the first phase of the COVID-19 pandemic in Greece.

## Methods

### Data Acquisition

The Social Feed Manager, an open source software (George Washington University Libraries) for harvesting data from social media [24], was used for the creation of the study data set. The study period was from January 25, 2020 to June 30, 2020. The collection was performed via Social Feed Manager using the filter-streaming application programming interface of Twitter. The search terms that were used for this purpose were selected from trending Twitter hashtags, identified at the beginning of the pandemic following a similar approach to another well-known data set [25]. The exact search keywords were as follows: *coronavirus, #coronavirus, SARS virus, #SARSvirus, #SARS2020, #SARS2, SARS-CoV, sars cov, SarsCov, #SarsCov, severe acute respiratory coronavirus, severe acute respiratory syndrome, #WuhanCoronavirus, #WuhanSARS, Wuhan Coronavirus, Wuhan SARS, 2019-nCoV, 2019 nCoV, #2019nCoV, 2019nCoV, COVID-19, #COVID19, COVID19.*

### Data Filtering

The collected tweets initially involved original tweets, retweets, quote tweets, and reply tweets in various languages. However, for this study, we considered only tweets that met the following inclusion criteria:

1. The language of the tweets was English.
2. The place where the tweets was made or the location of the user who created the tweets was Greece. Greece was specified using the following keywords: Greece, Hellas, Ellada, Ελλάδα, Ελλάς, Ελλαδα, and Ελλας.
3. The type of tweets was original or retweet. This selection was performed because the emotions of the users may be expressed not only in tweets written by themselves but also in tweets written by others, which the users decided to retweet.

### Emotional Analysis

#### Approach

The emotional analysis of the tweets that satisfied the inclusion criteria was achieved using a deep learning approach that performs better by utilizing recurrent neural networks on sequences of characters and not on sequences of words [26]. The character-based trained recurrent neural network models of this approach are available online on GitHub [26]. In this study, emotional epidemiology tools such as the 6 basic emotions (ie, joy, sadness, disgust, fear, surprise, and anger) based on Paul Ekman’s classification [27] were adopted. Joy is classified as a positive emotion, while the remaining 5 emotions are classified as negative; this concept was applied here. The deep learning approach was used to characterize each tweet by multiple emotions by counting them per day and presenting the proportion of each emotion per day during the study period. Daily, monthly, and phasic approaches were included in this investigation, and the emotions were summarized as negative, positive, or neutral as well.

#### Odds Ratio Calculation Between Phases

Three phases were defined: before lockdown, during lockdown, and after lockdown. The odds ratio (OR) of each emotion at each phase was calculated. The following formula was used:

OR= (a/c)/(b/d)

where a=specific dominant emotion X in phase Y, c=total emotions in phase X – specific dominant emotion X in phase Y, b=specific dominant emotion X in phase Z, d=total emotions in phase X – specific dominant emotion X in phase Z.

#### Emotional Retweet Network Graph Analysis

The monthly distribution of every emotion retrieved in retweets was represented in networks; neutral emotions were included. A retweet network is a directed weighted graph, where nodes represent Twitter accounts and edges represent the retweet relations. Subsequently, the graph t was transformed to its projection onto the users that retweeted, which is a well-known method for compressing the information of network graphs [28]. As a projection method, we applied the Ochiai coefficient (also known as cosine similarity) [29]. In the projected graph, only users who performed the retweets and not users who wrote the initial tweets are shown. The Force Atlas 2 layout in Gephi [30] was used so as to visualize the projected graph for the entire study period. The final step was to visualize the dominant emotion of the users per month (based on retweets only) in a projected graph by using an appropriate color palette.

#### Key Time Points of Analysis

We set 3 key time points (February 26, 2020 when the first COVID-19 case was diagnosed in Greece; March 23, 2020, when the lockdown was imposed; and May 4, 2020, when isolation measures were discontinued) that divided our target period into 4 subperiods: (1) before disease prevalence, (2) from first case until personal isolation measures, (3) lockdown subperiod, and (4) after lockdown subperiod. These subperiods were evaluated separately and comparatively.

## Results

### Corpus Statistics

**Figure 1:**
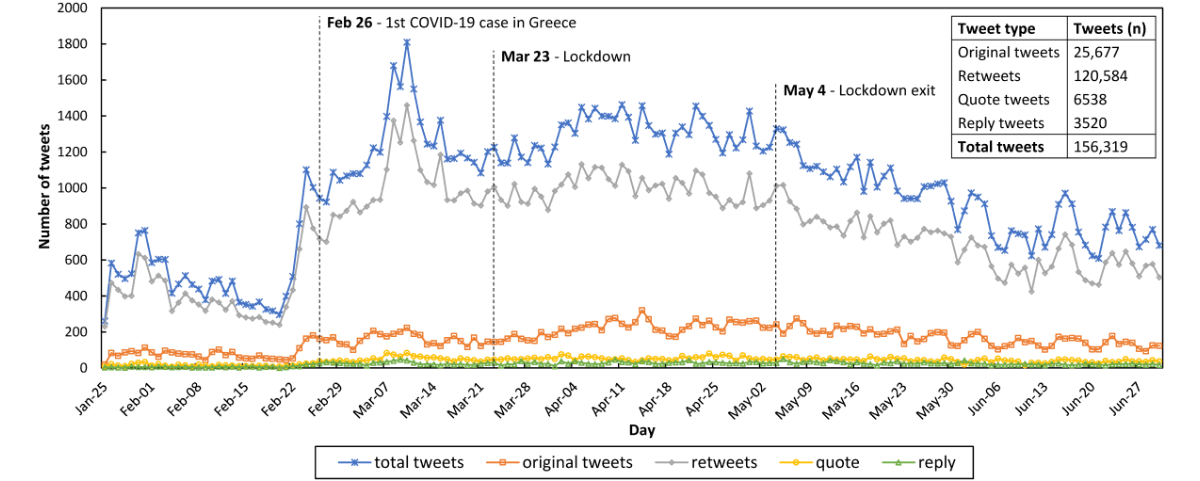
Daily account of English tweets by Greek Twitter users during the first phase of the COVID-19 pandemic in 2020.

We identified 529,694,030 tweets globally in the time period of interest. The number of COVID-19–related tweets that had been circulated during the first half of 2020 (January 25 to June 30, 2020) in Greece was 156,319. These tweets originating from Greece were produced by 12,994 unique Twitter accounts. The daily account of the dominant emotions during the study subperiods and the entire period are presented in Figure 1. However, in our emotional analysis, we included only original tweets and retweets, as only these types of tweets express the real feelings and agreement of the users with the text messages. Thus, the emotional analysis was performed on 146,261 tweets generated by 12,328 Twitter accounts.

### Emotional Analysis Results

The ORs and 95% CIs of each emotion at each phase were calculated and are presented in Table 1.

**Table 1:** Odds ratio (OR) and 95% CI of the basic emotions before, during, and after the lockdown in the first phase of the COVID-19 pandemic in Greece.^a^

Tweet type and comparisons		Joy, OR (95% CI)	Fear, OR (95% CI)	Anger, OR (95% CI)	Sadness, OR (95% CI)	Surprise, OR (95% CI)
**All tweets^b^**						
	BL/DL	0.739 (0.703-0.776)	1.067 (1.027-1.108)	1.128 (0.914-1.393)	1.127 (1.031-1.232)	1.095 (1.063-1.128)
	DL/AL	0.89 (0.852-0.93)	1.089 (1.048-1.132)	1.303 (1.036-1.638)	1.159 (1.056-1.274)	1.023 (0.993-1.053)
	BL/AL	0.657 (0.626-0.69)	1.162 (1.118-1.209)	1.47 (1.17-1.847)	1.307 (1.19-1.435)	1.12 (1.087-1.154)
**Original tweets**						
	BL/DL	0.659 (0.602-0.722)	1.307 (1.176-1.454)	0.901 (0.448-1.812)	0.964 (0.723-1.284)	1.257 (1.163-1.359)
	DL/AL	0.908 (0.844-0.978)	1.114 (1.004-1.236)	2.108 (0.986-4.506)	1.201 (0.918-1.572)	1.066 (0.99-1.148)
	BL/AL	0.599 (0.548-0.655)	1.457 (1.309-1.621)	1.899 (0.832-4.332)	1.158 (0.862-1.555)	1.34 (1.24-1.449)
**Retweets**						
	BL/DL	0.829 (0.781-0.88)	1.022 (0.981-1.065)	1.132 (0.907-1.413)	1.125 (1.024-1.237)	1.059 (1.026-1.094)
	DL/AL	0.911 (0.861-0.963)	1.077 (1.033-1.122)	1.224 (0.962-1.557)	1.144 (1.035-1.265)	1.008 (0.976-1.041)
	BL/AL	0.755 (0.712-0.802)	1.101 (1.055-1.148)	1.386 (1.093-1.758)	1.287 (1.166-1.421)	1.068 (1.034-1.103)

^a^BL: before lockdown; DL: during lockdown; AL: after lockdown.

^b^Original tweets and retweets.

**Figure 2:**
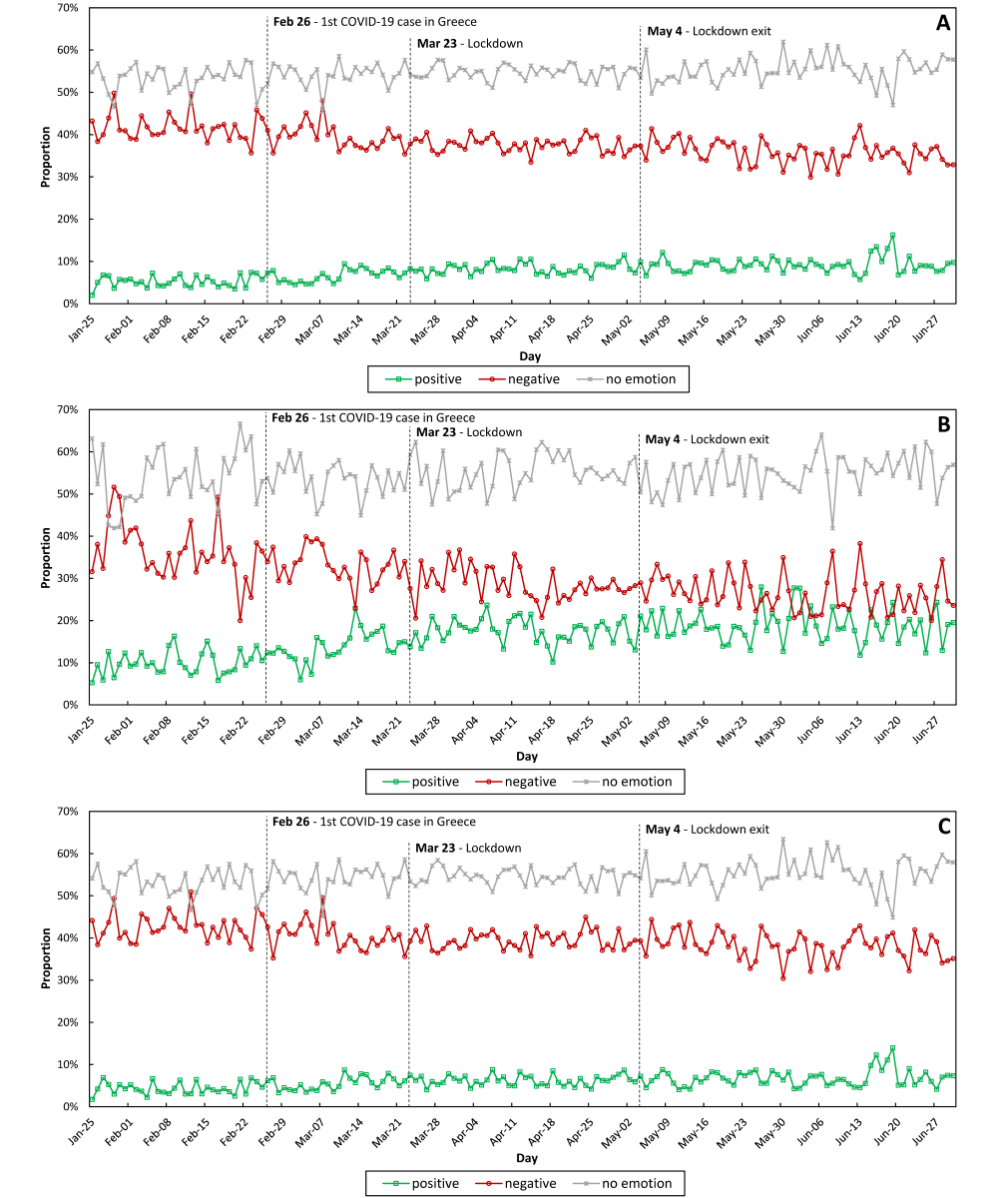
Daily distribution of positive, negative, and neutral emotions in (A) all tweets (ie, original and retweets), (B) original tweets, and (C) retweets.

Figure 2 depicts the distribution of positive, negative, and neutral tweets (original and retweets) per day during the study period. This plot shows an increasing trend of positive emotions from 5.42% on average in February 2020 to 9.28% in June 2020. Furthermore, the negative emotions showed a downward trend from 41.17% to 35.19% on average during the same months. The trend of percentage of neutral tweets varied from 46% to 62% of the total tweets in our analysis.

**Figure 3:**
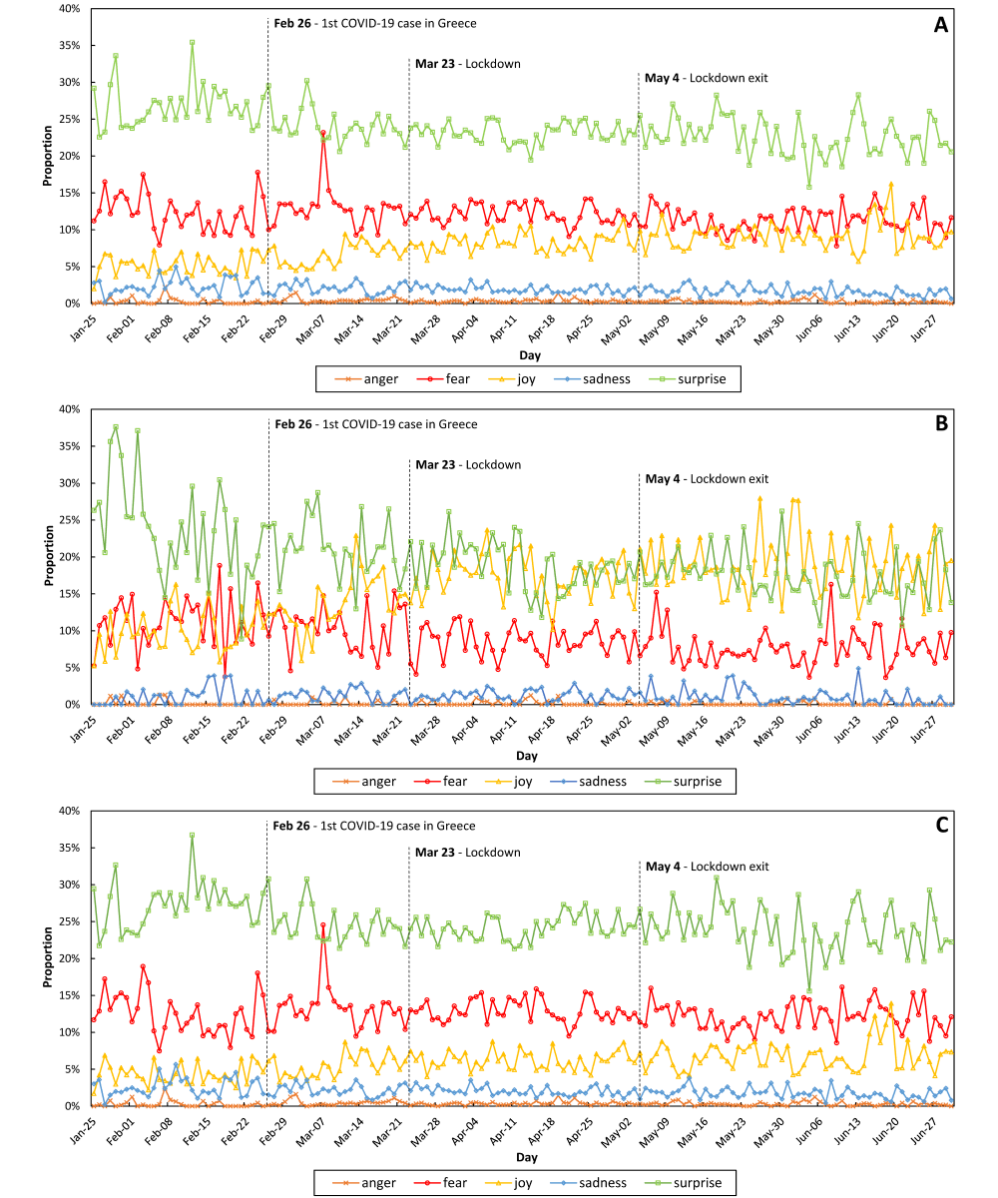
Daily distribution of the basic emotions in (A) all tweets (ie, original and retweets), (B) original tweets, and (C) retweets.

Figure 3 depicts the daily distribution of the basic emotions based on Ekman’s classification. In the included plots, the emotion of disgust was absent, as it was not detected in any tweet of our data set. The emotion of surprise was dominant during the entire study period, with an exception on March 7, 2020, showing overall a decreasing trend (27% on average in February 2020 to 22% in June 2020). Fear was ranked second, with some peaks in late February and early March showing an overall downward trend. In contrast, joy showed an increasing trend from 5.42% on average in February 2020 to 9.28% in June 2020.

### Emotional Graph Analysis Results

**Figure 4:**
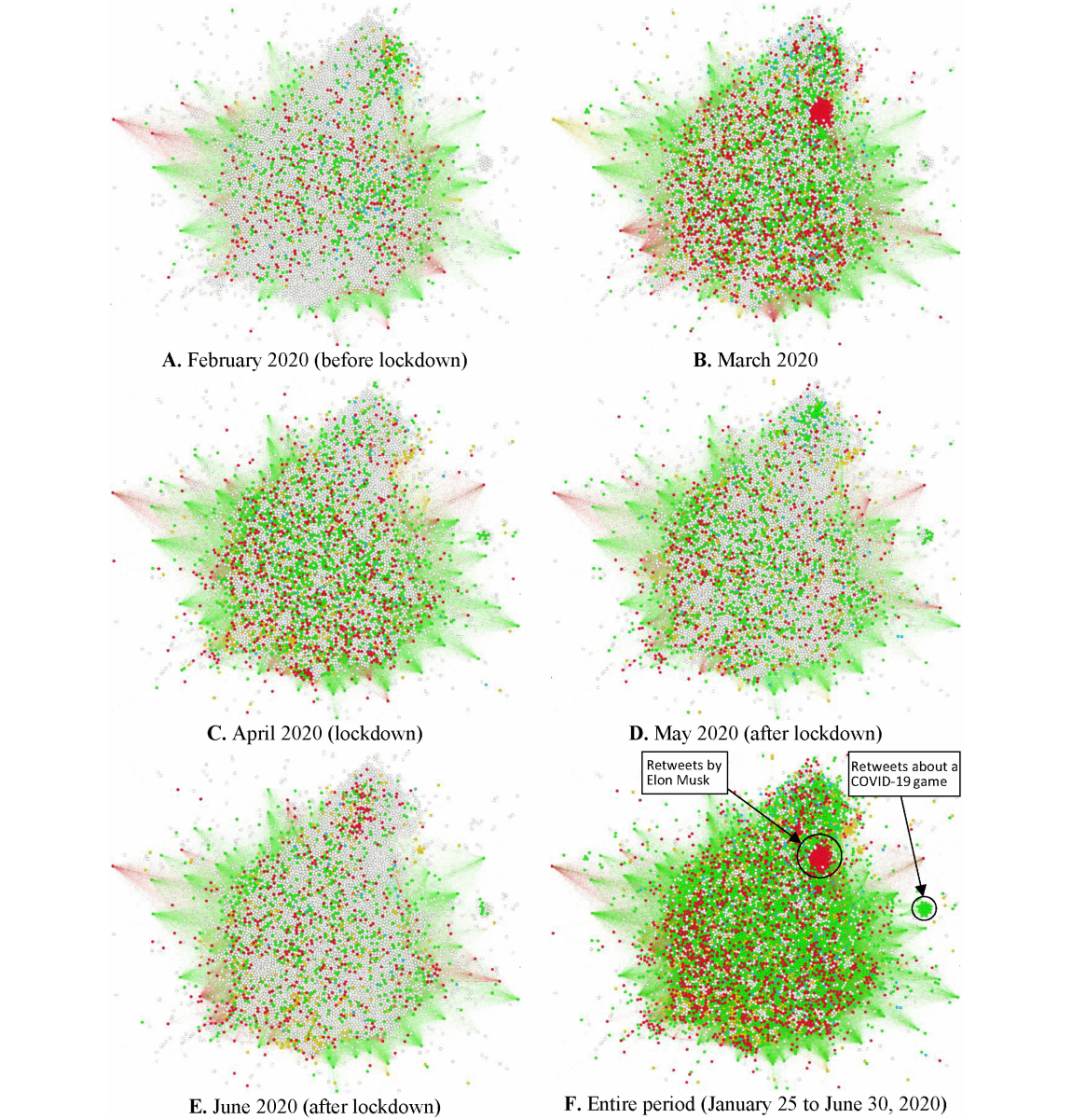
A force-directed visualization of Twitter accounts projection using Ochiai coefficient in the graph of retweets network, showing the dominant emotion of each account. White: neutral emotion; orange: anger; red: fear; yellow: joy; blue: sadness; green: surprise.

The monthly distribution of emotions of each Twitter account in the “retweets” network is presented in Figure 4. The nodes of this network represent Twitter accounts that performed retweets, the edges show the relations among the accounts, and the distance among the nodes shows how close these accounts were as it regards the retweets from the same source accounts. Figures 4A-4E show the dominant emotion every month on the same retweet network. Figure 4B illustrates a high increase in fear for many users, which, however, decreased in the ensuing months. In addition, the emotion of surprise, as identified in all subfigures, was high in users throughout the study period. Figure 4F presents the dominant emotion of each user for the entire period. Indicatively, we present 2 representative cases: (1) the spread of fear by a community of users that retweets messages originating from a unique source and (2) a community that retweets messages about a game to fight COVID-19.

## Discussion

### Overview of This Study

Emotional contagion has long been recognized in epidemiology [31-33], literature [34-38], politics [39], and the arts [40]. It does not need personal (vis-à-vis) contact, as the limbic system is intended to recognize and interpret nonverbal cues of “others” via empathy processes [41]. More importantly, it has been established that the social media body may influence and even fashion massive moods and opinions [15]. Scientific evidence suggests that positivity and negativity are two sides of the same coin [42]. Furthermore, social media platforms, which are actually an indispensable accessory of daily social/business/personal life, spread both information and misinformation and may influence the behavior of individuals and communities [43].

### Principal Results and Comparison With Prior Work

This work discusses the basic emotions of Greek populations expressed on Twitter during the first stage of the COVID-19 pandemic. Twitter is an open-source social media, where users may access and “retweet” (meaning reproduce) any message of anyone without being one of his/her “friends” or “followers.” These features make Twitter a critical pool of data for emotional, public and community health, and epidemiology evaluations [22,44]. The methodology followed is state-of-the-art and Twitter-specific [26], while the method suggested by Lwin et al [22] was not an open-source software. We identified 3 major events (first COVID-19 case imported from Italy on February 26, 2020; lockdown initiation on March 23, 2020; and lockdown end on May 4, 2020) that characterized this first flow and set 4 subperiods accordingly (before local epidemic onset, before lockdown, during lockdown, and after lockdown subperiods). We evaluated these subperiods separately and for comparisons. Our analysis showed that the (total) “all tweets” flow was modified by retweet tendencies (Figure 1). The same was identified in a previous work examining more nations [22].

Uncertainty due to SARS-CoV-2 may trigger emotional distress, anxiety, and even depression, as observed before in previous epidemic flows [45]. Our analysis was based on Paul Ekman’s classification of 6 basic emotions: joy, surprise, fear, disgust, anger, and sadness. In the literature, several theories have been proposed, suggesting various models, that is, circumflex model suggested by Russel [46], dimensional models [47], vector model [48], Pleasure-Arousal-Dominance model [49], positive-negative activation model [50], and the groupings model, that is, Parrott’s grouping [51]. However, scientists have not reached consensus on the constraints or the underlying neurobiological mechanisms of emotions and experiences [52]. To this end, including trust or temperament, which has been associated with the efficacy of public health measures [13,14], to the evaluation of our information would be too ambitious and involving possible bias or arbitrary interpretations in terms of Twitter-derived limited information. We opted for the Paul Ekman classification because (1) it is an established method, (2) it is simple and feasible for Twitter-specific shortfall of information, and (3) other classifications, including gradient emotions [53], need to provide more information to be valid. Such an attempt would be dependent on a different methodology—beyond the reach of our social media investigation.

Disgust was not identified in any tweet or retweet in our pool of data. Surprise was the first reaction to the broadcast news. Unlike other emotions that increased or decreased before and after the lockdown, the surprise emotion increased during the lockdown, as the pandemic was unexpected and the turmoil and information were taken with a surprise mainly during the isolation when daily professional concerns or social distractions ceased. Surprise may lead to an “acute stress response” [54] and may even mask fear [55]. Coronaphobia is a new term describing the persistent fear induced by the SARS-CoV-2 contagion [56]. Research of previous epidemics suggested that the frequency of such phobias fluctuates and may originate from intolerance of uncertainty, personal susceptibility to concern and fear, and individual disease vulnerability [33,57]. Fear is a basic instinct of survival, bringing about more composite emotions such as anxiety or depression or situations such as insomnia. Insomnia prevalence was found similar to the fear trend in Greek health care workers [17,20]. In our previous work, we calculated the worldwide contagion probability of the first COVID-19–induced fear on Twitter as high as 0.288 [22,23], while the total fear probability in the social media platforms was as high as 0.322 [17]. In this analysis, fear ranked second, while the fear odds ratio increased by 0.307 in the original tweets, 0.55 in retweets, and 0.22 in total tweets. Furthermore, this analysis showed that the fear effect size was greater in the original tweets when we compared the time periods before and after the lockdown, probably because individuals faced a new reality that interrupted their regular way of life. Fear levels increased in retweets and “all tweets” as well. The isolation strained family bonds and exposed individuals to a storm of information and misinformation as well as to a looming uncertain future [58]. During a relevant period in China, more than half of the survey responders rated the psychological impact of the pandemic as moderate-to-severe [59]. Xenophobia was not identified in our pool of data unlike misinformation-induced (infodemic) fear. More explicitly, in our network analysis, we identified clusters of fear in retweets of tweets originating from a unique source (Figure 4B and 4F). This cluster was limited in March (Figure 4B), moderating the tendency of the entire period (Figure 4F).

Societal uncertainties such as those observed during epidemics may trigger fear and anger in persons and communities [22,60]. A survey targeting the mental health of Greek children and adolescents in April to May 2020 identified significant mental effects of lockdown on the children, which was moderated by increased family conflicts, parental mental history, parental unemployment/lack of opportunity to web-based occupational activity, or children’s physical history record [61]. An adolescent-targeted Italian study showed that psychopathological history combined with “worries about infection” is linked to anxiety, while psychopathological history combined with female gender triggers depression [62]. An adult-targeted survey conducted in April 2020 in Greece showed significant variations in fear and anxiety levels, which were definitely age or (female) gender-dependent [10]. The difference is fundamental as reported by Plutchik’s Wheel of Emotions: fear originates from circumstances whereas anger from persons [17,22,63]. The same was reflected in the differences observed. In our analysis, anger was the most influential emotion during the isolation period. Anger was greater between the time period of lockdown and immediately after the lockdown (odds ratio 2.108, 95% CI 0.986-4.506) and was detected in the subdata of the original tweets. Importantly, this was the strongest feeling expressed amid all comparisons between time periods or emotions (Table 1). The literature referring to previous epidemics such as Ebola and SARS associated lockdown with anger, establishing that anger increased the risk of confusion, mental disease (such as posttraumatic stress disorder), unexpected behaviors [64-66], and suicides [62,67,68]. The anger-related wide confidence intervals calculated (during versus after lockdown) reflected those observations and the individuality of responsiveness. The measures of surveillance may be responsible for this: people experienced boredom owing to the duration of the restriction and fear of the infection peril in combination with unfavored preventive measures and resistance of the community to comply with them. This is a common phenomenon identified and explained since antiquity by Thucydides as mentioned above [5,7]: citizens often refuse to accept reality owing to the panic, thereby neglecting any rule suggested by sanitary authorities. Such an attitude of “others” results in anger against those that want to comply and survive. Anger also masks stress preceding a “flight or fight” reaction [54].

The effectiveness of public health measures depends upon compliance and is related to anger and fear levels. Greeks complied absolutely to the government’s early-applied restriction of quarantine during the first phase of the pandemic; the levels of joy were decreased during the lockdown in comparison to that before the lockdown owing to the social isolation. However, the fluctuation in the joy levels followed that in the surprise levels and presented a delay of 1 day after fear spiked; probably, the actuality imposed levels of joy each time. The current globalization and the modern fast-paced life have distracted persons from interperson ability to be at peace with oneself. The levels of joy did not decrease dramatically in Greece: citizens grasped the opportunity to cherish familial bonds, enjoy hobbies, or stress solidarity (a virtue deeply rooted in Greek mentality but forgotten in the past recent decades). The government’s tough measures taken early prevented the havoc of death incidence that other countries experienced [14]. For the Greeks, this was a virtual reality seen in news broadcasts. Additionally, stress is often masked by joy [42], especially in Greek mentality and mood. Joy seeks to retain homeostasis and balance the allostatic load of stress or other negative emotions. Thus, joy in the original tweets did not fluctuate violently as anger did. Joy is the only positive emotion in the Ekman classification [27]. As seen in Figure 2, the positive emotions illustrated reflect purely joy. Pure joy is reflected in a cluster related to a virtual game (Figure 4F). The virtual entertainment was a privilege and a double-edged sword in this pandemic. Although people profited from this virtual service, it failed to prevent anger as described above.

Sadness caused by the loneliness during lockdown and the sense of frailty in view of the increasing death rates in other countries is illustrated in Figures 3 and 4. Although stress levels were increased mostly after the lockdown compared to that before the lockdown period, in terms of effect sizes, the daily prevalence seemed rather unaffected.

Neutral emotions were consistent (as shown in Figures 2A-2C) amid total original tweets and retweets. The latter, expressed in a monthly trend in the networks, are included in Figure 4. Neutral emotions represented mainly “business” tweets, that is, announcements and reports of various organizations such as the government. The levels of neutral emotions in the tweets were higher than those of positive and negative emotions. Daily fluctuations in neutral emotions were more intense after the exit from the lockdown because the lifting of the imposed restrictions after the lockdown was stepwise and modest, depending on preventive measures. The exit from the lockdown coincided with a period that is critical for professionals, students, families, businesses, that is, the onset of summer, as tourism is one of the pillars of Greek economy and a season-dependent sector for Greece.

Positive (joy, in fact) emotion fluctuation was rather flat in the retweets as well as in the total tweets, but the positive emotions definitely increased after the exit from the lockdown. The feeling of relief from the imposed restrictions and the socioeconomic restart was dominant at the time. However, negative emotions (summarizing anger, surprise, fear, sadness, stress) manifested an increasing tendency through the 3 phases. The same was observed in the American population [69]. This is attributed to the nature of these emotions. Primary or secondary negative emotions follow the epidemic flow progress. As mortality rates follow fatality increase, negative emotions intertwine positive ones even in a country modestly affected by SARS-CoV-2 during the first phase of the COVID-19 pandemic (like Greece).

### Limitations and Strengths of This Study

This study is limited to the 6 basic emotions classified by Paul Ekman. The other classifications were not evaluated. Another limitation is the location (Greece) and the time period of interest, which pertains to the first COVID-19 wave (January 2020 to June 2020). Future research should focus on the second and third waves. The value of this work extends to the effectiveness of public health rules and therapeutic interventions, as emotions may influence treatment progress in chronic, infectious, and psychiatric diseases [33,68,70]. In a different context, emotions would also mediate prosocial behaviors and intentions [14], where trust (not included, though, in the 6 basic emotions suggested by Paul Ekman’s classification) is the key player/target point for public health planning effectiveness and efficacy. The latter was revealed to be mediated by moral principles and behavioral intentions as well [14].

### Conclusions

In conclusion, a combined approach of emotions in Twitter may contribute to defining the epidemiology of emotions in general or during epidemics. Of all the emotions in the English tweets of Greek Twitter users, “surprise” dominated in the initial period, while fear and anger dominated during the lockdown in the first stage of the COVID-19 pandemic. Surprise is a manifestation of the “acute stress response to the newly emerging threat in citizens’ (users’) personal lives.

## Abbreviations

OR: Odds ratio
